# Contrast-Enhanced Ultrasound in the Differentiation between the Most Common Benign Parotid Gland Tumors: A Systematic Review and Meta-Analysis

**DOI:** 10.3390/jcm11247360

**Published:** 2022-12-12

**Authors:** Marta Rogalska, Lukasz Antkowiak, Anna Kasperczuk, Wojciech Scierski

**Affiliations:** 1 Faculty of Medicine, Medical University of Warsaw, 02-091 Warsaw, Poland; 2 Department of Pediatric Neurosurgery, Medical University of Silesia in Katowice, 40-752 Katowice, Poland; 3 Faculty of Mechanical Engineering, Institute of Biomedical Engineering, Bialystok University of Technology, 15-351 Bialystok, Poland; 4 Department of Otorhinolaryngology and Oncological Laryngology, Faculty of Medical Sciences in Zabrze, Medical University of Silesia in Katowice, 41-800 Zabrze, Poland

**Keywords:** ultrasonography, salivary gland, pleomorphic adenoma, Warthin’s tumor, perfusion

## Abstract

Recently, contrast-enhanced ultrasound (CEUS) has become a promising tool in distinguishing benign from malignant parotid gland tumors. However, its usefulness in differentiating various benign parotid tumors has not been determined so far. This study aimed to systematically review the literature to determine the utility of CEUS in the preoperative differentiation between pleomorphic adenomas (PAs) and Warthin’s tumors (WTs) of the parotid gland. PubMed, Embase, and Cochrane were searched for English-language articles published until 21 July 2022. Fifteen studies were included. On CEUS examination, a significantly greater percentage of PAs displayed heterogeneous enhancement texture compared to WTs. Contrarily, the enhanced lesion size, the enhancement margin, and the presence of the enhancement rim did not differ significantly between the entities. Significantly longer normalized mean transit time (nMTT) and time to peak (TTP) were observed in PAs. Contrarily, the mean values of area under the curve (AUC) and time from peak to one half (TPH) were significantly higher for WTs. Due to the considerable overlap among the qualitative CEUS characteristics of PAs and WTs, the reproducible, investigator-independent quantitative CEUS measurements have a greater potential to distinguish PAs from WTs, which might influence the selection of an appropriate management strategy.

## 1. Introduction

Parotid gland tumors constitute approximately 80.0% of all salivary gland neoplasms [1]. Most of them (75–80%) are benign, with pleomorphic adenomas (PAs) and cystadenolymphomas (Warthin’s tumors; WTs) being the most frequent entities and accounting altogether for up to 93% of all benign parotid gland tumors [2,3]. Their preoperative differentiation remains crucial for selecting an appropriate management strategy. PAs have been reported to carry a 2–25% risk of malignant transformation. Additionally, since their recurrence rate increases after an inadequate surgical procedure, such as enucleation, at least a partial parotidectomy is recommended. Contrarily, the vast majority of WTs do not recur, and only anecdotal reports describing its malignant transformation exist in the literature [4,5,6]. Therefore, the current trend in the management of WTs is to minimize the extent of resection using partial parotidectomies or, when feasible, extracapsular dissections, whereas in elderly multimorbid patients with surgical contraindications, a conservative approach with active surveillance might also be selected [7,8].

Currently, B-mode ultrasonography (US) remains a widely used tool in the diagnostic workup of parotid gland tumors. However, due to the considerable overlap in ultrasonographic features between PA and WT, including the vascular characteristics on Doppler sonography, US parameters alone do not allow a reliable differentiation between these neoplasms [9]. Similarly, ultrasound elastography (USE) was proposed as a potentially effective tool in distinguishing PAs and WTs [10,11]. Nevertheless, growing evidence indicates inconsistencies between the studies due to the meaningful overlap in elasticity between WTs and PAs (which may display a wide range of stiffness), which currently precludes their unequivocal differentiation by means of USE [12,13,14].

Routinely performed MRI, especially the analysis of T2-weighted images and apparent diffusion coefficient (ADC) values, might be helpful in the preoperative diagnostic process of benign parotid tumors [15,16]. However, some WTs might occasionally be misdiagnosed as PAs when a cystic component is present [17].

Fine-needle aspiration cytology is a commonly used first-line tool for pathological diagnosis of parotid gland lesions. However, numerous studies have emphasized its limitations, such as a high rate of false-negative results and poor accuracy in differentiating various types of neoplasms [18]. In turn, core needle biopsy carries a significant risk of facial nerve injury and might be complicated by local tumor seeding, postoperative hematoma, and infections due to the violation of the previously intact mass capsule during the procedure [19].

In recent years, contrast-enhanced ultrasound (CEUS) has been extensively explored as a novel ultrasound modality in differentiating salivary gland tumors. Intravenously injected contrast medium consists of gas bubbles stabilized with a layer of phospholipid or galactose. Due to their small size (1 to 5 µm on average), microbubbles can circulate through the capillary system. This facilitates the assessment of parenchymal perfusion and lesional microvascularity, particularly in echo-free areas, therefore, enabling the exclusion of cystic and necrotic compartments. The purely intravascular contrast agent does not leak in the interstitial spaces [20,21] and allows continuous flow evaluation (real-time perfusion imaging). Moreover, CEUS yields measurable and comparable perfusion kinetics [22], providing objective quantitative data.

In the Sultan et al. meta-analysis [23], the ability of perfusion-related CEUS parameters to distinguish benign parotid gland tumors from malignancies has been reported. However, the articles assessing CEUS usefulness in discriminating various benign parotid neoplasms have described inconsistent findings. Therefore, the purpose of the present study was to systematically review the literature to determine the utility of CEUS in the differentiation of PAs and WTs of the parotid gland.

## 2. Materials and Methods

### 2.1. Study Guidance

The review was conducted according to the PRISMA 2020 (Preferred Reporting Items for Systematic Reviews and Meta-Analyses) guidelines [24]. The study protocol was registered with the International Platform of Registered Systematic Review and Meta-analysis Protocols (INPLASY) under the number INPLASY2022120042 [25].

### 2.2. Search Strategy and Criteria

The PubMed, Embase, and Cochrane databases were searched by two authors (M.R. and L.A.) independently for English-language full-text papers published from inception until 21 July 2022. The comprehensive electronic search strategies included terms for parotid gland tumors (“parotid” OR “parotid gland” OR “parotid gland lesion” OR “parotid lesion” OR “parotid neoplasm” OR “parotid cancer” OR “parotid carcinoma” OR “parotid tumour” OR “parotid tumor” OR “parotid mass” OR “salivary” OR “salivary gland” OR “salivary gland lesion” OR “salivary lesion” OR “salivary neoplasm” OR “salivary cancer” OR “salivary carcinoma” OR “salivary tumour” OR “salivary tumor” OR “salivary mass”) AND terms for CEUS (“contrast-enhanced ultrasound” OR “contrast enhanced ultrasound” OR “CEUS” OR “microbubbles ultrasound”). After duplicates removal, all studies were screened by two authors (M.R. and L.A.) independently, based on the title and the abstract. Inclusion criteria comprised clinical studies evaluating differential diagnosis of benign parotid tumors using CEUS. Publications with an unrelated topic, conference papers, review articles, case reports, commentaries, technical notes, and letters to the editor were excluded. Additionally, the reference lists in all preselected articles were screened for further relevant papers. Any discrepancies between the researchers were discussed until a consensus was reached.

### 2.3. Eligibility Criteria

The study was eligible if it qualitatively and/or quantitatively evaluated CEUS-derived data in patients with PA or WT of the parotid gland.

### 2.4. Data Extraction

From the included studies, the following data were extracted: first author and publication year, study design, number of benign parotid gland tumors (PA/WT), reference standard, contrast agent, a time considered for analysis following contrast administration, the region of interest (ROI) selection, tumor characteristics on CEUS images, and the assessed CEUS parameters.

### 2.5. Quality Assessment

Two reviewers (M.R. and L.A.) independently assessed the quality of the eligible studies according to the Quality Assessment of Diagnostic Accuracy Studies (QUADAS-2) tool [26]. Any discrepancies between the reviewers were resolved through discussion until a consensus was reached.

### 2.6. Evaluation of Qualitative CEUS-Derived Data

The qualitative analysis of CEUS-derived parameters comprised the assessment of the following aspects: (1) the enhancement intensity (hyper-, iso-, or hypoenhancement) of the tumor compared to the surrounding salivary gland tissue; (2) the enhancement texture (homogenous or heterogeneous); (3) the enhancement margins (well-defined or ill-defined); (4) the presence of an enhancement rim; (5) the enhanced lesion size (increased or unchanged) compared with the lesion area before enhancement; (6) the presence of echo-free areas (corresponding to contrast-free perfusion areas in the tumor after the enhancement); (7) the perfusion pattern (centripetal, meaning a perfusion pattern from the periphery into the center of the lesion, or non-centripetal, in case of perfusion from the center of the lesion to the periphery or a diffuse central pattern); (8) the wash-in pattern (referring to whether the tumor starts to enhance earlier, simultaneously, or later than the surrounding normal gland); and (9) the wash-out pattern (referring to whether the lesion starts to fade earlier, simultaneously, or later than the surrounding normal gland).

### 2.7. Evaluation of Quantitative CEUS-Derived Data

**Figure 1:**
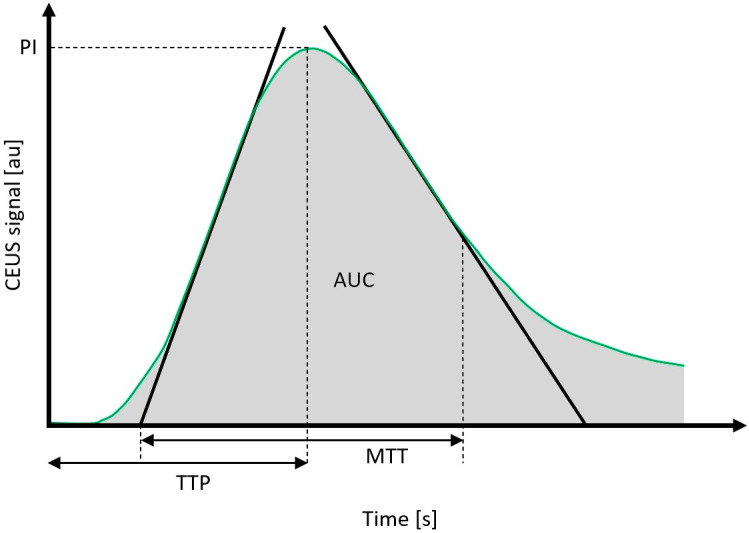
Quantitative time-to-intensity CEUS curve parameters. CEUS, contrast-enhanced ultrasound; au, arbitrary units; s, seconds; PI, peak intensity; AUC, area under the curve; TTP, time to peak; MTT, mean transit time.

The quantitative evaluation of the CEUS-derived parameters involved the analysis of: (1) rise time (RT, in seconds), representing the time during which the time-intensity curve (TIC) increases from the starting point to 50% of the peak value; (2) peak intensity (PI, in dB), representing the maximum signal intensity measured in the selected ROI; (3) mean transit time (MTT, in seconds), representing the time during which the curve decreases from the starting point to 50% of the PI; (4) normalized mean transit time (nMTT, in seconds), representing the mean transit time normalized by circumjacent parotid tissue and expressed in ratios; (5) area under the curve (AUC, in arbitrary units), representing the area under the entire time-intensity curve; (6) time from peak to one half (TPH, in seconds), representing the time from peak to half of the absolute increment; (7) time to peak (TTP, in seconds), representing the time from the contrast agent injection to the maximum intensity of the contrast agent signal; (8) normalized time to peak (nTTP, in seconds), representing the time to peak normalized by circumjacent parotid tissue and expressed in ratios; (9) rising slope (RS, in dB/s), calculated using the formula (peak intensity-baseline intensity)/rise time; (10) wash-in-rate (WiR, in arbitrary units), describing the rate of change of contrast agent inflow; and (11) wash-in-perfusion-index (WiPI, in arbitrary units), defined as wash-in area under the curve divided by RT. The graphical representation of the selected CEUS-derived parameters is presented in Figure 1.

Overall data, including benign parotid tumors other than PAs and WTs, were not used for the quantitative analysis. Additionally, if data on a specific tumor type (e.g., PA) comprised lesions located in salivary glands other than the parotid gland, they were excluded from the quantitative analysis.

### 2.8. Statistical Analysis

As the studies included in the meta-analysis came from different centers and covered slightly different populations, the summary was performed by applying a random effect. As the end result, the mean value with a 95% confidence interval (CI) was chosen. Statistical heterogeneity in the studies was assessed using the I2 statistics. For values above 50%, further analysis was performed to identify the source of the heterogeneity, allowing the inclusion of homogeneous studies only. The mean values were then compared between the groups (PA and WT) with a series of *t*-tests for the two means in order to determine the significance. Additionally, qualitative CEUS features of PAs and WTs available throughout the articles were compared using the Chi2 test. Differences were considered significant at *p* < 0.05. The analysis was performed using Statistica 13.3 (StatSoft Polska, Krakow, Poland) and PQStat 1.8.4 (PQStat Software, Poznan, Poland) software.

## 3. Results

### 3.1. Study Selection

**Figure 2:**
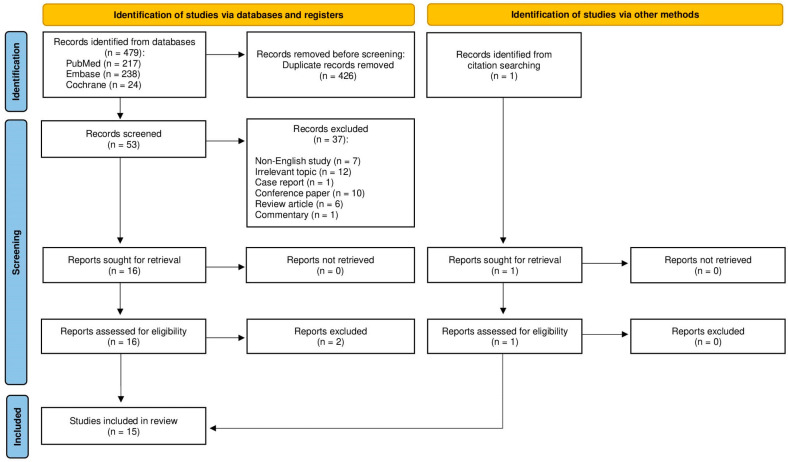
PRISMA flowchart of the medical database search strategy.

The literature search yielded 479 articles, including 217 from PubMed, 238 from Embase, and 24 from Cochrane. After the removal of 426 duplicate records, 53 studies were screened. Seven non-English studies and 12 articles with an irrelevant topic were excluded, as well as 1 case report, 10 conference papers, 6 review articles, and 1 commentary. Out of 16 studies assessed for eligibility, 2 were found ineligible—the first one did not determine if the analyzed salivary gland tumors were located in the parotid or the submandibular gland, and the second one did not provide any specific qualitative and quantitative CEUS-derived data. The remaining 14 articles were found eligible. After the identification of one relevant publication from the reference lists, a total of 15 studies were included in the further analysis. Figure 2 shows the entire literature selection process.

### 3.2. Study Characteristics

Nine studies [7,27,28,29,30,31,32,33,34] described the qualitative CEUS features of PAs and WTs. Three of them [29,31,34] qualitatively analyzed PAs and WTs located in the parotid or submandibular gland. The remaining six articles [7,27,28,30,32,33] provided data on PAs and/or WTs located solely in the parotid gland. Thirteen studies [7,27,28,29,30,31,32,33,35,36,37,38,39] assessed the values of quantitative CEUS parameters. Complete study characteristics are presented in Table 1.

### 3.3. Study Heterogeneity

Following the evaluation of the 12 statistics, studies by Bozzato et al. [33] and Saito et al. [39] showed values ≥ 50%; thus, they were excluded from the meta-analysis. Therefore, the meta-analysis of each parameter is based on homogeneous studies, with 12 statistics values < 50%.

### 3.4. Study Quality

**Figure 3:**
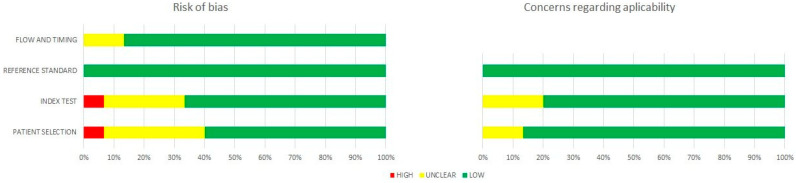
Summary of the quality evaluation of the included studies by means of QUADAS-2 tool.

The assessment by the QUADAS-2 tool revealed a moderate or excellent quality of the included studies, as shown in Figure 3. Nevertheless, several methodological shortcomings contributing to bias still existed. 

Two studies [29,30] with inappropriate exclusion criteria and six studies [7,29,30,34,35,37] without a consecutive or random sample of enrolled patients might have introduced potential selection bias. Concerning the index test, five studies [27,33,35,37,39] could increase the risk of bias because they did not report the interpretation of the CEUS assessment without the knowledge of the histopathological examination. Additionally, two studies [31,34] might have enlarged the risk of bias with respect to flow and timing because the described patients underwent either core needle biopsy or surgical resection for the final pathological diagnosis. However, the interpretation of the reference standard in all included studies was regarded as carrying a low risk of bias.

### 3.5. Qualitative CEUS-Derived Data

Qualitative analysis of CEUS-derived parameters was performed in nine studies [7,27,28,29,30,31,32,33,34]. The majority of PAs showed hypo- or isoenhancement [7,34], heterogenous enhancement texture [7,27,29,31,34], simultaneous or late wash-in pattern (“slow in”) [7,34], and early wash-out pattern (“fast out”) [7,34]. The presence of central non-enhancing areas (pseudocysts and necrosis) was observed significantly more often within PAs than in the WT group [7,28]. Additionally, a trend towards higher marginal perfusion (centripetal perfusion pattern) in PAs was reported [33,34].

Contrarily, most WTs were characterized by hyperenhancement [7,34], homogenous enhancement texture [7,28,29,31,34], early wash-in pattern [7,34] (“fast in”), and simultaneous or late wash-out pattern (“slow out”) [7,34]. Moreover, several authors described a trend toward hypervascularization of WTs compared to PAs [30,33] and a trend toward non-centripetal perfusion pattern in WTs [32,33,34].

Both PAs and WTs showed mostly well-defined enhancement margins [7,29] and unchanged enhanced lesion size [7,29,34]. Although Jiang et al. [29] reported that significantly more PAs showed rim enhancement compared to adenolymphomas (*p* < 0.05), this finding was not confirmed by other studies [7].

The enhanced lesion size (increased or unchanged), the presence of an enhancement rim, the enhancement margin (well-defined or ill-defined), and the type of enhancement (homogeneous or heterogeneous) were the only qualitative CEUS features available throughout the included articles. Based on this data, our meta-analysis revealed statistically significant differences in the enhancement texture (homogenous vs. heterogeneous) between PAs and WTs (*p* < 0.001). A significantly greater percentage of PAs displayed heterogeneous enhancement texture (94.99%) compared to the WT group (26.47%). Contrarily, the enhanced lesion size, enhancement margin, and presence of an enhancement rim did not differ significantly between the PA and WT groups (*p* = 0.566, *p* = 0.848, and *p* = 0.548, respectively).

### 3.6. Quantitative CEUS-Derived Data

The meta-analysis included the assessment of the following parameters: RT, MTT, nMTT, AUC, TPH, TTP, nTTP, RS, WiR, and WiPI. The PI was not included in the meta-analysis due to the heterogeneity of the evaluated studies. The results of the quantitative analysis of the CEUS parameters are summarized in Table 2.

Significantly higher values of nMTT were observed in the PA group (1.45; 95% CI, 1.31–1.59) than in the WT group (0.62; 95% CI, 0.51–0.74) (*p* = 0.002). TTP displayed significantly higher values in the PA group (26.92; 95% CI, 22.46–31.39) compared to the WT group (16.92; 95% CI, 16.04–17.72) (*p* = 0.001). The mean values of AUC in the WT group (107.99; 95% CI, 46.28–169.71) were significantly higher than those in the PA group (66.26; 95% CI, 57.66–74.87) (*p* < 0.001). TPH exhibited significantly higher values in the WT group (57.30; 95% CI, 40.54–74.06) compared to the PA group (46.29; 95% CI, 31.44–61.14) (*p* = 0.001).

Based on the findings obtained from 24 patients (13 with PAs, 11 with WTs), Klotz et al. [37] described significantly higher values of WiR and WiPI in the WT group (16.3; 95% CI, 13.23–18.83, and 44.6; 95% CI, 37.20–52.00, respectively) than in the PA group (3.26; 95% CI, 2.51–4.01, and 8.0; 95% CI, 6.87–9.13, respectively) (*p* < 0.001). Moreover, in the Knopf et al. study [30] including 16 patients (8 with PAs, 8 with WTs), nTTP was significantly shorter in WTs (0.74; 95% CI, 0.67–0.81) than in PAs (1.46; 95% CI, 1.17–1.75) (*p* < 0.001). In turn, based on the data of 88 patients (54 with PAs, 34 with WTs), Yan et al. [7] found significantly higher values of MTT in the WT group (63.90; 95% CI, 40.00–87.80) than in the PA group (36.75; 95% CI, 24.93–48.57) (*p* < 0.001). Contrarily, in the same Yan et al. study [7], no significant differences between PAs and WTs were noted in the RT and RS values.

## 4. Discussion

The introduction of ultrasound contrast agents has created opportunities to facilitate a differential diagnosis between various benign and malignant parotid lesions. In recent years, CEUS has been established as a valuable diagnostic tool, providing a microvascular perfusion analysis in solid tumor tissue with well-documented hepatic and non-hepatic applications [41]. Its non-invasive nature, high resolution, and favorable level of patient acceptance, as well as the lack of radiation hazard, have caused CEUS to widen the diagnostic spectrum of US modalities. As the microbubble contrast agent is primarily excreted through the respiratory tract and metabolized in the liver, it can be administered in patients with severe renal function impairment [42]. Additionally, due to the strong safety profile of the contrast medium with a low risk of adverse events, contrast injections can be repeated, which enables monitoring of the dynamic performance of the contrast agent over time [42].

### 4.1. Qualitative CEUS-Derived Data

The low enhancement pattern (hypo- or isoenhancement) reported in most PAs might be attributed to their development from benign glandular epithelial tumors, which is characterized by slow growth and sparse vascular distribution [7]. Furthermore, the presence of abundant and unevenly dispersed mucinous, cartilaginous, and/or hyaline mesenchymal components mixed with epithelial tissue is presumably the reason for PAs’ heterogeneous enhancement texture [2,7]. The diverse arrangement of different PAs’ morphological segments, with a non-uniform, predominantly marginal, and tortuous blood vessel distribution, might explain the centripetal and “slow in” perfusion pattern of PAs [7,34].

Various reports [2,43] have suggested that WTs originate from ectopic lymphatic tissue in the salivary gland and exhibit a dense microvascular distribution, causing a marked hyper-enhancement similar to that in inflammatory lymph nodes. Additionally, the uniform and dense microscopic arrangement of intralesional cellular components (lymphocytes and glandular epithelial cells) with sparse interstitial space result in a homogenous enhancement texture of WTs [2,34].

Most PAs and WTs displayed the typical CEUS features of benign salivary gland tumors, i.e., well-defined enhancement margins and unchanged enhanced lesion size [7,29,34]. However, on rare occasions, PAs exhibited blurred enhancement margins, presumably due to the active cell growth and partially incomplete tumor capsule [34]. Moreover, the increased enhanced lesion size encountered in several WTs might stem from their location in the superficial part of the salivary gland, which affected the observation through the lateral acoustic shadow and the inability to focus on the lesion located too close to the probe [29,34].

Despite the statistically significant difference between PAs and WTs in terms of enhancement type, the overlap in other qualitative CEUS characteristics (the enhanced lesion size, the enhancement margin, and the presence of the enhancement rim) might indicate the insufficient reliability of the descriptive benign parotid tumors evaluation. The qualitative CEUS assessment is, to a certain extent, operator-dependent, rendering the data obtained in this way prone to interobserver variability. While a certain combination of multiple qualitative CEUS characteristics might be suggestive of a specific benign parotid tumor type, the definitive diagnosis cannot be reached based solely on qualitative CEUS-derived data. Its limited discriminatory ability demonstrates the necessity of combining descriptive evaluation with the analysis of objectively acquired parameter values. 

### 4.2. Quantitative CEUS-Derived Data

Our meta-analysis revealed significantly higher values of AUC in WTs, indicating higher perfusion intensity in these lesions compared to PAs. Moreover, Welkoborsky et al. [32] demonstrated that the medial (more distant from the ultrasound transducer) parts of both tumor types (ROIs 4 through 6) showed higher AUC values compared to lateral (located more superficially, closer to the ultrasound transducer) lesion parts (ROIs 1 through 3). Additionally, the AUC displayed significantly higher values in all ROIs in WTs compared to the corresponding ROIs in PAs [32]. The differences between ROIs throughout the lesions were higher in WT than PA, indicating a perfusion heterogeneity in both tumor types, which was nevertheless more pronounced in WTs [32]. 

In our meta-analysis, the nMTT in PAs (1,45; 95% CI, 1.31–1.59) was significantly longer than in WTs (0,62; 95% CI, 0.51–0.74), which corresponds to a delayed perfusion pattern in PAs (nMTT > 1 s) and reflects enhanced perfusion kinetics in WTs (nMTT < 1 s) [38]. Similarly, the significantly shorter TTP in the WT group compared to the PA group corresponds to the faster wash-in rate of the contrast agent through the WT ROIs. Additionally, the significantly higher values of TPH in WTs reflect a slower wash-out rate in this tumor type compared to PAs, corresponding to the rich capillary network characteristic of WTs.

The results of our meta-analysis demonstrate that contrast agent kinetic analysis in PAs and WTs of the parotid gland offer statistically significant investigator-independent variables. The described widely reproducible quantitative parameters (AUC, nMTT, TTP, TPH) appear more accurate in the differentiation of PAs and WTs of the parotid gland than the use of qualitative CEUS-derived data. The objective qualitative measurements could presumably be utilized to determine the urgency and the required extent of the surgery, particularly in the case of PAs, which carry a much higher risk of malignant transformation than WTs. Additionally, the reliable identification of WTs through quantitative CEUS assessment could reduce operative risk by suggesting a less aggressive surgical procedure, or even an observational approach in elderly patients with severe comorbidities.

Nevertheless, further large cohort studies providing a repeatable quantitative analysis of other CEUS parameters (PI, MTT, nTTP, RT, RS, WiR, WiPI) are highly warranted in order to determine the actual usefulness of this promising technique in the routine management of patients with benign parotid gland tumors.

### 4.3. Limitations

Our systematic review has highlighted the limited number of studies investigating the use of CEUS in the differential diagnosis of benign parotid tumors. Due to the low incidence rate of benign parotid neoplasms other than PAs and WTs and the lack of literature aiming at differentiating them from PAs and WTs, they were not included in our meta-analysis. Further studies assessing their CEUS characteristics are highly warranted.

A different number of ROIs and their various placements for perfusion analysis throughout the included studies constitutes another major limitation of our research. Only in one study [31] did the authors perform an evaluation with a systematic ROI distribution throughout the entire tumor tissue. In most other articles, the perfusion parameters values were measured in a single ROI, particularly within the highly perfused part of the lesion. Additionally, the diversity in the tracking duration of the contrast inflow after its injection (ranging from 30 s to 180 s) renders the comparison of the results difficult. The significant heterogeneity between research protocols necessitates caution in interpreting both the presence and the lack of statistically significant differences between PAs and WTs. Future numerous, satisfactorily homogenous studies are required to precisely define the extent of CEUS utility and reliability in managing patients with benign parotid tumors.

## 5. Conclusions

Despite the scarcity of literature, recent reports imply the potential utility of CEUS in the differentiation between PAs and WTs of the parotid gland. The significantly greater heterogeneous enhancement texture of PAs compared to WTs might reflect the microstructural differences between these entities. However, the overlap in other descriptive CEUS characteristics might indicate the insufficient accuracy of differentiating PAs and WTs based solely on qualitative CEUS assessment. Significantly longer nMTT and TTP in PAs and significantly higher values of AUC and TPH in WTs demonstrate the potential of quantitative CEUS assessment in distinguishing PAs from WTs. Nevertheless, the limited number of studies investigating the use of CEUS in the differential diagnosis of benign parotid tumors renders caution in interpreting our data. Future large prospective studies including standardized CEUS-based assessment are highly warranted to precisely define the extent of CEUS reliability in the management of patients with benign parotid gland tumors.

## Figures and Tables

**Table 1:** Characteristics of included studies.

First Author (Year)	No. of Tumors (PAs/WTs)	Reference Standard	Contrast Agent	ROI	Time Considered for Analysis	Assessed Quantitative CEUS Parameters	Qualitative CEUS Findings
Badea (2013) [27]	12 (12/0)	histopathology	SonoVue	whole tumor	120 s	TTP	CEUS uptake pattern in PAs was variable due to the displacement of the tumor vessels towards periphery (homogenous, 4/12; inhomogeneous, 6/12).
Bozzato (2007) [33]	81 (51/30)	histopathology	SonoVue	Area within the tumor decided by the user	90 s	PI, TTP, WiT, WiV	PAs showed a presence of distal echo enhancement and a trend to higher marginal perfusion, whereas WTs presented a tendency towards higher blood supply and manifested a marked central perfusion trend.
Fischer (2010) [28]	18 (9/9)	histopathology	SonoVue	Vascularized area within the tumor	60 s	AUC, TTP	Contrast enhancement differed between PAs and WTs. In the PA group: an increase in echogenicity mainly in the tumor periphery; no enhancement of the center in 7/9 PAs; central non-enhancing areas corresponding to pseudocysts and necrosis. In the WT group: small nodular peripheral defects and fairly homogeneous central enhancement in 5/9 WTs; well-demarcated small cystic areas; central enhancement was absent in only 2/9 cases
Guiban (2021) [40]	44 (16/28)	histopathology	SonoVue	Area within the tumor decided by the user, and adjacent parotid tissue	120 s	NA	NA
Guo (2021) [35]	98 (98/0)	histopathology	SonoVue	Highly perfused area within the tumor	120 s–180 s	TTP, Peak, RBV, RBF, ∆Si_max_, Si_mean_	NA
Jiang (2020) [29]	52 (28/24)	histopathology	SonoVue	Area within the tumor with avid enhancement, and surrounding parotid tissue	90 s	TTP, PI, TPH	PAs were significantly more heterogeneous than WTs. Significantly more PAs showed rim enhancement compared to WTs.
Klotz (2013) [36]	32 (17/15)	histopathology	SonoVue	At the centre of solid tumor tissue, and surrounding parotid tissue	90 s (first 30 s)	AUC, MMT, TTP, ∆Si_max_	NA
Klotz (2014) [37]	24 (13/11)	histopathology	SonoVue	Area within the tumor decided by the user	90 s (first 30 s)	AUC, PI, RT, WiR, WiPI	NA
Knopf (2012) [30]	16 (8/8)	histopathology	SonoVue	Area within the tumor decided by the user, normalized by surrounding parotid tissue	NA	nMTT, nTTP	CEUS visualized poor perfusion in PAs and apparent hyperperfusion in WTs.
Mansour (2017) [38]	137 (64/73)	histopathology	SonoVue	Area within the tumor decided by the user, normalized by surrounding parotid tissue	NA	nMTT	NA
Saito (2021) [39]	23 (10/13)	histopathology	Sonazoid	Area within the tumor decided by the user	120 s	TTP, AUC, Grand	NA
Wang (2022) [34]	58 (36/22)	histopathology	SonoVue	Area within the tumor with abundant blood flow, and surrounding parotid tissue	90 s	NA	The differences between PAs and WTs were statistically significant in terms of enhancement degree, enhancement pattern, enhancement homogeneity, and wash-in pattern. Most PAs showed simultaneous or later wash-in, inhomogeneous hypo- or isoenhancement and centripetal enhancement pattern. Contrarily, most WTs displayed earlier wash-in, homogeneous hyperenhancement, and non-centripetal enhancement pattern.
Wei (2013) [31]	132 (62/70)	histopathology	SonoVue	Area within the remarkable perfusion area of the tumor, and surrounding parotid tissue	180 s	AUC, TTP, PI	All PAs showed heterogeneous enhancement, mainly corresponding to the CEUS type 2b, whereas all WTs displayed diffuse homogeneous enhancement (representing CEUS type 1).
Welkoborsky (2022) [32]	67 (31/36)	histopathology	SonoVue	8 standardized areas distributed throughout the entire tumor	NA	AUC, TTP, Peak	Perfusion pattern significantly differed between PAs and WTs. No visible perfusion was found in 42% of PA and in 11% of WT, a peripheral (centripetal) perfusion pattern in 19.5% and 22%, respectively, and a centrifugal perfusion pattern in 6.5% and 25%, respectively.
Yan (2021) [7]	88 (54/34)	histopathology	SonoVue	Area within the solid tumor tissue, and surrounding parotid tissue	120 s	AUC, MTT, RT, PI, TPH, TTP, RS	The majority of WTs showed high homogeneous enhancement with a “fast in” and “slow out” pattern. Contrarily, most PAs displayed low heterogenous enhancement and presented with an echo-free area, and a pattern of “slow in” and “fast out”.

Legend: PAs, pleomorphic adenomas; WTs, Warthin’s tumors; ROI, region of interest; CEUS, contrast-enhanced ultrasound; TTP, time to peak; nTTP, normalized time to peak; AUC, area under the curve; MTT, mean transit time; nMTT, normalized mean transit time; NA, non-applicable; RT, rise time; WiR, wash-in-rate; WiPI, wash-in-perfusion-index; PI, peak intensity; TPH, time from peak to one half; RS, rising slope; RBV, regional blood volume; RBF, regional blood flow; ∆Si_max_, maximum signal intensity; Si_mean_, mean signal intensity; Grand, curve gradient of wash-in; Peak, the increase of signal intensity from the baseline to maximum of intensity; WiT, wash-in time; WiV, wash-in velocity; s, seconds.

**Table 2:** Comparison of quantitative CEUS characteristics between PAs and WTs.

Feature	PAs Mean (95% CI)	WTs Mean (95% CI)	*p* Value
Rise time (RT) [s]	4.08 (2.56–5.60)	4.36 (2.49–6.23)	0.444
Mean transit time (MTT) [s]	36.75 (24.93–48.57)	63.90 (40.00–87.80)	<0.001
Normalized mean transit time (nMTT) [s]	1.45 (1.31–1.59)	0.62 (0.51–0.74)	0.002
Area under curve (AUC)	66.26 (57.66–74.87)	107.99 (46.28–169.71)	<0.001
Time from peak to one half (TPH) [s]	46.29 (31.44–61.14)	57.30 (40.54–74.06)	0.001
Time to peak (TTP) [s]	26.92 (22.46–31.39)	16.92 (16.04–17.72)	0.001
Normalized time to peak (nTTP) [s]	1.46 (1.17–1.75)	0.74 (0.67–0.81)	<0.001
Rising slope (of wash-in curve) (RS) [dB/s]	2.42 (1.34–5.76)	1.28 (0.84–1.72)	0.052
Wash-in-rate (WiR)	3.26 (2.51–4.01)	16.3 (13.23–18.83)	<0.001
Wash-in-perfusion-index (WiPI)	8.0 (6.87–9.13)	44.6 (37.20–52.00)	<0.001

Legend: CEUS, contrast-enhanced ultrasound; PAs, pleomorphic adenomas; WTs, Warthin’s tumors; CI, confidence interval.

## Data Availability

The data generated during this study are available within the article. Datasets analyzed during the current study preparation are available from the corresponding author on reasonable request.
